# A Descriptive Comparative Pilot Study: Association Between Use of a Self-monitoring Device and Sleep and Stress Outcomes in Pregnancy

**DOI:** 10.1097/CIN.0000000000000958

**Published:** 2022-11-28

**Authors:** Jennifer Auxier, Milad Asgari Mehrabadi, Amir M. Rahmani, Anna Axelin

**Affiliations:** **Author Affiliations:** Department of Nursing Science, The University of Turku, Finland (Ms Auxier); Department of Electrical Engineering and Computer Science, University of California Irvine (Mr Asgari Mehrabadi); Department of Computer Science and School of Nursing, University of California Irvine (Dr Rahmani); and Department of Nursing Science, The University of Turku, and Department of Obstetrics and Gynaecology, Turku University Hospital and Faculty of Medicine, University of Turku, Finland (Dr Axelin).

**Keywords:** Behavioral changes, Pregnancy, Self-care, Sleep, Wearable sensors

## Abstract

Pregnancy is a challenging time for maintaining quality sleep and managing stress. Digital self-monitoring technologies are popular because of assumed increased patient engagement leading to an impact on health outcomes. However, the actual association between wear time of such devices and improved sleep/stress outcomes remains untested. Here, a descriptive comparative pilot study of 20 pregnant women was conducted to examine associations between wear time (behavioral engagement) of self-monitoring devices and sleep/stress pregnancy outcomes. Women used a ring fitted to their finger to monitor sleep/stress data, with access to a self-monitoring program for an average of 9½ weeks. Based on wear time, participants were split into two engagement groups. Using a linear mixed-effects model, the high engagement group showed higher levels of stress and a negative trend in sleep duration and quality. The low engagement group showed positive changes in sleep duration, and quality and experienced below-normal sleep onset latency at the start of the pilot but trended toward normal levels. Engagement according to device wear time was not associated with improved outcomes. Further research should aim to understand how engagement with self-monitoring technologies impacts sleep/stress outcomes in pregnancy.

Pregnancy is a time of physical and emotional changes. Many pregnant persons experience pain, discomfort, and bodily changes that have been linked to sleep disturbances that can increase the prenatal experience of stress.^1,2^ Sleep disturbances are common during pregnancy because of hormonal and physiological changes and manifest as insomnia or sleep fragmentation.^3,4^ Antenatal stress and sleep disturbances have been linked to increased likelihood of preterm birth.^5,6^ Sleep disturbances have also been associated with incidence of stillbirth and growth and weight restrictions.^6^ Because of a low frequency of contact visits during the early antenatal period, nonmedical concerns such as sleep disturbances and stress in early pregnancy have been historically left out of antenatal care.^2^ It is now possible to monitor sleep quality and mental health states linked to stress during pregnancy using eHealth modalities.^7–10^ A self-monitoring technology that provides insights into actual sleep patterns and stress responses could support pregnant persons toward building a greater bodily awareness in between antenatal clinic visits.^11,12^

If pregnant persons are given access to sleep and stress data collected using three-dimensional accelerometer, gyroscope, and biomarker signals including photoplethysmogram (PPG) and electrocardiogram through wearable recording devices and Bluetooth technology, they might be able to engage in lifestyle self-monitoring that stimulates their commitment to manage their sleep disturbances and levels of stress. Increasing pregnant persons' commitment (behavioral engagement)^13^ over time might influence the quality of care.^14^

Pregnant persons use eHealth modalities to remind them about important issues during pregnancy when so much is already on their minds.^15^ Self-monitoring technology is being used by persons living with chronic conditions such as diabetes and multiple sclerosis in order to motivate and support behavioral engagement in self-care.^16,17^ These eHealth users experienced greater condition awareness and benefited from setting goals toward health behaviors.^16,17^ The use of self-monitoring technologies has raised some concerns about user overburden due to feelings of needing to perform after viewing their personal data and experiences of decision fatigue related to access and choice of self-monitoring devices.^17–19^ Participation in self-care could be enhanced with the use of wearable devices and viewing of personal data; however, associations between behavioral engagement and health outcomes are not well understood.

It is common for pregnant persons to experience sleep disturbances during pregnancy, and the effects of such disruptions to normal rest and sleep have been shown to be positively correlated with fatigue, childbirth fear, and anxiety.^20^ Although sleep and stress are important clinical problems, we know that personalized antenatal health promotion coaching for sleep and stress is often difficult because of the common inaccuracy of visit self-reports of stress, mental health states, and sleep quality.^21,22^ This results in an assessment gap at the time of clinic visits. In the past decade, self-monitoring of personal health data has made it possible to monitor sleep duration and quality, and the levels of stress during pregnancy in between visits.^12,23^ Although the assessment gap can be lessened with the use of these technologies, little is understood about the impact technological interventions of self-monitoring might have on behavioral engagement in self-care and subsequent quality of care and health outcomes. Previous studies investigating perinatal technological sleep self-monitoring have not investigated the association between behavioral engagement and health outcomes using multiple week collection of PPG signal sleep and stress parameters.^24,25^

## Self-monitoring and the Engaged User

Technological self-monitoring care processes are often aimed at motivating users to become engaged in their own self-care.^26–28^ Having access to wearable devices and personal sleep and stress data makes it possible for pregnant persons and healthcare providers to assess health without a greater use of health service resources related to in-person clinic visits.^2,29^ Although self-monitoring technology is gaining popularity in perinatal care in high- to middle-income countries, the associations between behavioral engagement in wearing devices and stress and sleep outcomes remain unclear. Studies examining the effectiveness of self-monitoring in pregnancy have revealed conflicting results; more should be investigated on the impact of self-monitoring activities on improved health outcomes.^8,9,30,31^

Wearable device monitoring and viewing personal data have shown to be highly effective and reliable for use in daily life of users and for research purposes.^11,23,32^ Self-monitoring modalities support the collection of data about stress levels, sleep duration, and quality.^12,23^ Changes over time can also be examined related to pregnant users' behavioral engagement of wearing devices (eg, wear time). The objective of this pilot study was to observe any associations between pregnant persons' behavioral engagement and changes in sleep duration, and quality and levels of stress. Behavioral engagement was measured using amount of wear time of the smart ring device (worn on the finger).

## METHODS

The pilot study investigated the implementation and demand of using a smart ring self-monitoring technology in a Finnish antenatal clinic.^33^ This study is one phase of a larger feasibility study examining engagement by pregnant persons in a perinatal eHealth program using self-monitoring and goal setting for physical activity, stress, and sleep in collaboration with their public health nurses. In the present pilot report, implementation and demand were examined by comparing the user groups according to their level of behavioral engagement (ie, wear time) and their trends in sleep duration and quality and levels of stress over the course of the pilot period.^33^

### Study Participants and Setting

Pregnant persons receiving care at one antenatal clinic in Southwest Finland were sampled using convenience sampling.^34^ Participants were enrolled in the study between March and August 2020 during their first or early second trimesters. Inclusion criteria included being 18 years or older, having access to a smartphone (Android or iOS), and having good literacy in Finnish and English languages. Six public health nurses were enrolled in the larger feasibility study; they received the smart rings and use of the wellness Web and smartphone applications (apps) at the start of the study to familiarize themselves with the use of the self-monitoring technology. Public health nurses agreed to participate in the larger feasibility study to act as testers and supports for the pregnant persons who used the self-monitoring and goal setting eHealth program. Participants were recruited during late first trimester of pregnancy or early to mid-second trimester to allow for a nurse visit schedule to include two or more visits prior to the end of the larger feasibility study.

### Pilot Use of Ōura Technology

Pregnant persons who consented to take part in the study received the wearable ring to be worn on their finger and access to the smartphone and Web ŌURA apps through Bluetooth pairing and anonymized user logins. The ŌURA technology is a commercially available wearable device to be worn on the finger. Version 2.0 was used in this study and was able to monitor sleep and stress data. The stress levels were interpreted from the recording of heart rate variability during sleep. All nighttime recordings can be uploaded to the smartphone and Web apps through a Bluetooth connection each day. Users could access the Web app to view more details on their data trends and download their own data if they wished. The smartphone app provided daily tips and feedback about best practices for maintaining low stress levels and sleep duration and quality. As a part of the larger feasibility study, pregnant participants were instructed to wear the ring as much as possible every day in ways that best suited them. They were instructed also to discuss use of the smart ring and apps, self-monitoring, and goal setting for physical activity, stress, and sleep with their public health nurse over the course of the study.

### Data Collection

Participants piloted the self-monitoring program for, on average, 9.5 weeks. The smart ring device has been tested and validated for the monitoring of sleep and heart rate variability data.^11,32,35^ Participants in our study recorded daily sleep and stress data whenever they wore the smart ring. Participants completed demographic, use and availability of technology, and health parameter survey data at baseline of the study period. Participants were informed that if the smart ring was uncomfortable or not recording well or the battery did not last between normal charging periods they had to contact the nurse researcher for assistance. The data were uploaded with Bluetooth pairing to the ŌURA cloud service supported by a data sharing and storing system provided through the ŌURA company.

#### Nonwear Time

A record is kept through the PPG signal detection of the smart ring indicating when the ring is being worn. Total minutes of nonwear time per day was recorded by the device and uploaded to the cloud storage through Bluetooth connection.

#### Sleep Duration and Quality

Total sleep time (TST) is a measure of duration of total sleep during the night.^4^ Sleep quality was measured in our study using sleep onset latency (SOL), waking after sleep onset (WASO), and sleep efficiency. Sleep onset latency is the time it takes to move from a fully wakeful state to a sleep state determined by polysomnography.^36^ The ŌURA ring 2.0 has been validated to measure this parameter using PPG signal and hand movement indicators (eg, accelerometer).^11^ Sleep onset latency is commonly experienced as equal to or less than 20 minutes.^36^ Waking after sleep onset was recorded in the length of time spent awake after sleep onset; this indicated how much sleep disturbance is experienced according to disrupted TST.^4^ Sleep efficiency was calculated by dividing TST by the sum of TST, SOL, and WASO.

#### Levels of Stress

The root mean square of successive differences (RMSSD) reflects the variance in heart rate beat-to-beat and is a primary time-domain measure for estimating the vagally mediated changes reflected in heart rate variability.^37^ Lower values of RMSSD are indicative of increased impact on the parasympathetic nervous system as a response to physiological stress exposure.^38^

### Statistical Analysis

#### Data Preprocessing

ŌURA smart ring provides the daily data summary for sleep and stress parameters in a structured format. We utilized Python 3.8 to parse these files and extract parameters we were interested in. Because ŌURA reports all the sleep events, we labeled the ones happening during nighttime and focused only on night sleep.

Descriptive statistics (means, ranges, and distribution of values) of participants demographic and questionnaire totals were organized and prepared for analysis using R for statistical analyses (version 3.6.1; R Core Team, Vienna, Austria).

#### Kernel Density Estimate Analysis

To cluster our subjects into high and low engagement groups, we extracted the nonwear time of the smart ring and looked at the normalized distributions and observed two groups of users based on the characteristics of the distributions. We leveraged kernel density estimation on the nonwear time to estimate such normalized distributions. Kernel density estimation is a useful nonparametric tool to estimate the distributions and helps to distinguish different clusters of data.^39^

#### Linear Mixed-Effects Model Analysis

To model the characteristics of the participants, a hierarchical linear mixed model was exploited for each of the high and low engagement groups (Supplemental Digital Content 1, http://links.lww.com/CIN/A201). Using this model, we were able to analyze the between-subject, within-subject, and overall trends. The single within-subject independent variable was the time (day), and the health outcomes related to sleep duration and quality and levels of stress were the dependent variables in this study.

### Ethics

Ethical approval was obtained by the ethics committee of the Hospital District of Southwest Finland prior to the start of the study (approval ID: ETMK Dnro: 1/1801/2020). Pregnant persons and public health nurses provided informed consent before participation in the larger feasibility study.

## FINDINGS

Six public health nurses and 20 pregnant women agreed to participate in the larger feasibility study. Finnish pregnant women joined the study during their first or early second trimesters. All women had low risk pregnancies at the start of the study, with one participant requiring bed rest later in the study period. Eighteen (90%) of the women were employed, and seven (35%) of the women experienced chronic illnesses outside of pregnancy.

Seventy percent of women in the study stated that their pregnancies negatively impacted their sleep quality (71.4%; n = 10 of high engagement group; 66.6%; n = 4 of low engagement group). All participants stated that they had an unlimited smartphone data plan to use in the study. We experienced some technical difficulties regarding appropriate smart ring sizes and faulty batteries; however, women received fast technical service and new smart rings within 24 hours of their reported concerns (Supplemental Digital Content 2, http://links.lww.com/CIN/A202).

### Engagement Measured by Wear Time: Kernel Density Estimate Analysis

**FIGURE 1:**
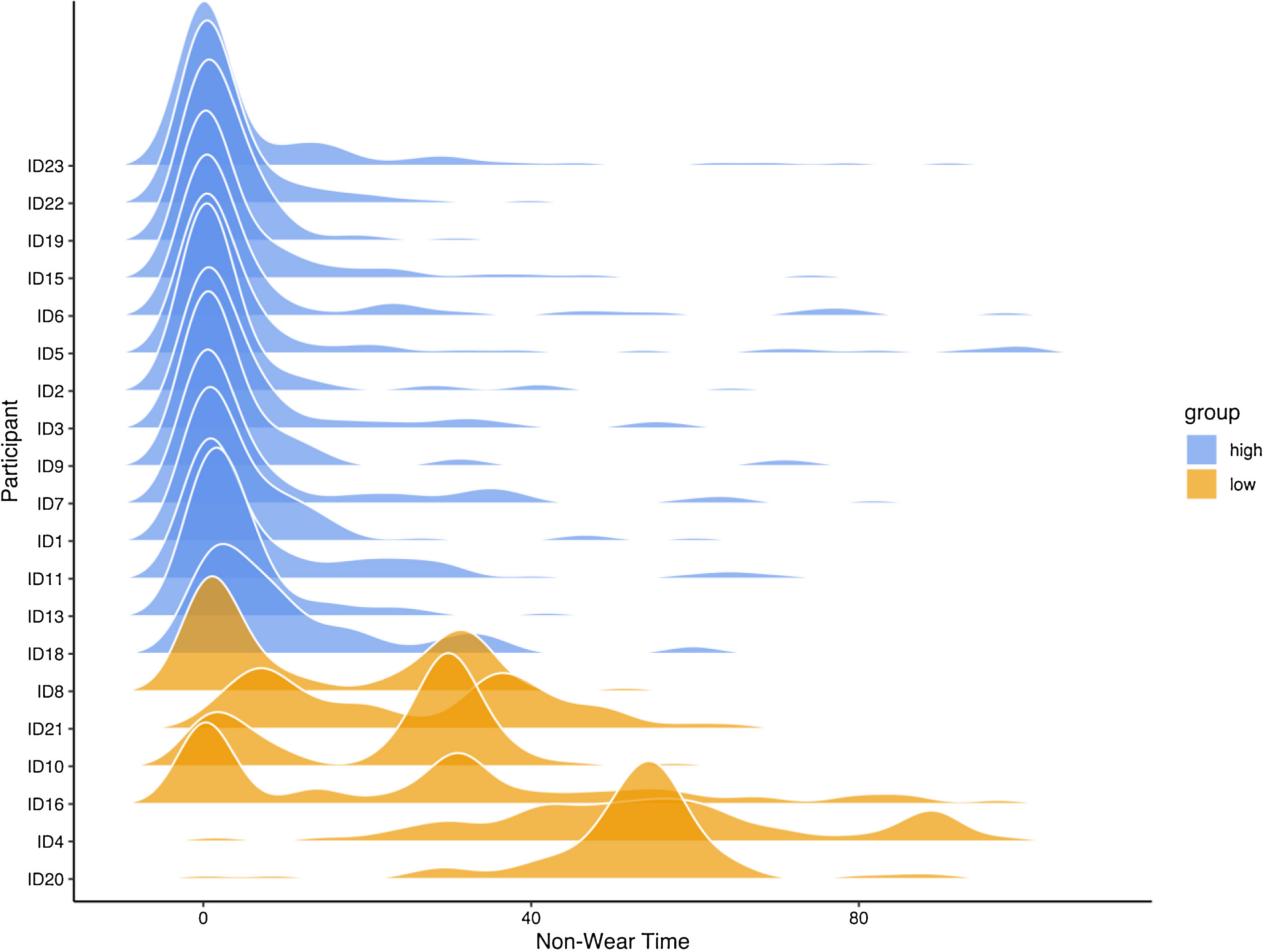
Distributions of user nonwear time. Values represent normalized values of nonwear time.

Women were spilt into high and low engagement group by clustering subjects with a normalized nonwear time less than 20% as high engagement group (n = 14; 70%) and the rest as low engagement group. Distributions according to participants can be seen in Figure 1. See Table 1 for background data according to user groups.

**Table 1:** Baseline Characteristics of Participating Pregnant Women According to Engagement Group

	High Engagement (n = 14)	Low Engagement (n = 6)
Age, mean (SD), year	32 (2.42)	29 (3.01)
Average pregnancy weeks (baseline)	15 + 4	15 + 3
Able to wear device at work,^a^ % (n)	92.85 (13)	16.66 (1)
Employed, % (n)	85.7 (12)	100.0 (6)
Body mass index, mean (SD), kg/m^2^	24.95 (17.43–31.64)	26.48 (20.96–39.84)
Nonwear time average, %	7.13	32.49
Medical condition,^b^ % (n)	28.6 (4)	50.0(3)
No. of children	One child = 6 Two children = 3 No children = 5	One child = 2 Two children = 1 No children = 3
Planned pregnancy, % (n)	85.7 (12)	100.0 (6)
Frequency of other app use in daily life	Daily = 8 Weekly = 5 Monthly = 1 Rarely = 0	Daily = 3 Weekly = 2 Monthly = 0 Rarely = 1
EPDS scores (baseline)	4.57 (2.90) Range, 1–12	3.50 (3.08) Range, 0–8
Perceived stress (baseline)	37.14 (5.14) Range, 29–44	37.67 (7.66) Range, 27–46
PRAQ-R (baseline)	5.57 (0.85) Range, 4–7	5.67 (0.52) Range, 5–6
Sense of coherence (baseline)	72.64 (5.62) Range, 62–81	77.00 (4.73) Range, 71–84

Abbreviations: EPDS, Edinburgh Postnatal Depression Scale; PRAQ-R, Pregnancy-related Anxiety Questionnaire-Revised.

^a^Women were unable to wear the ring on their fingers for health and safety reasons during working hours.

^b^Medical conditions included: migraines; asthma; hypothyroidism; ulcerative colitis; & endometriosis.

### Sleep Duration and Quality Changes Over Time According to Engagement Groups

**FIGURE 2:**
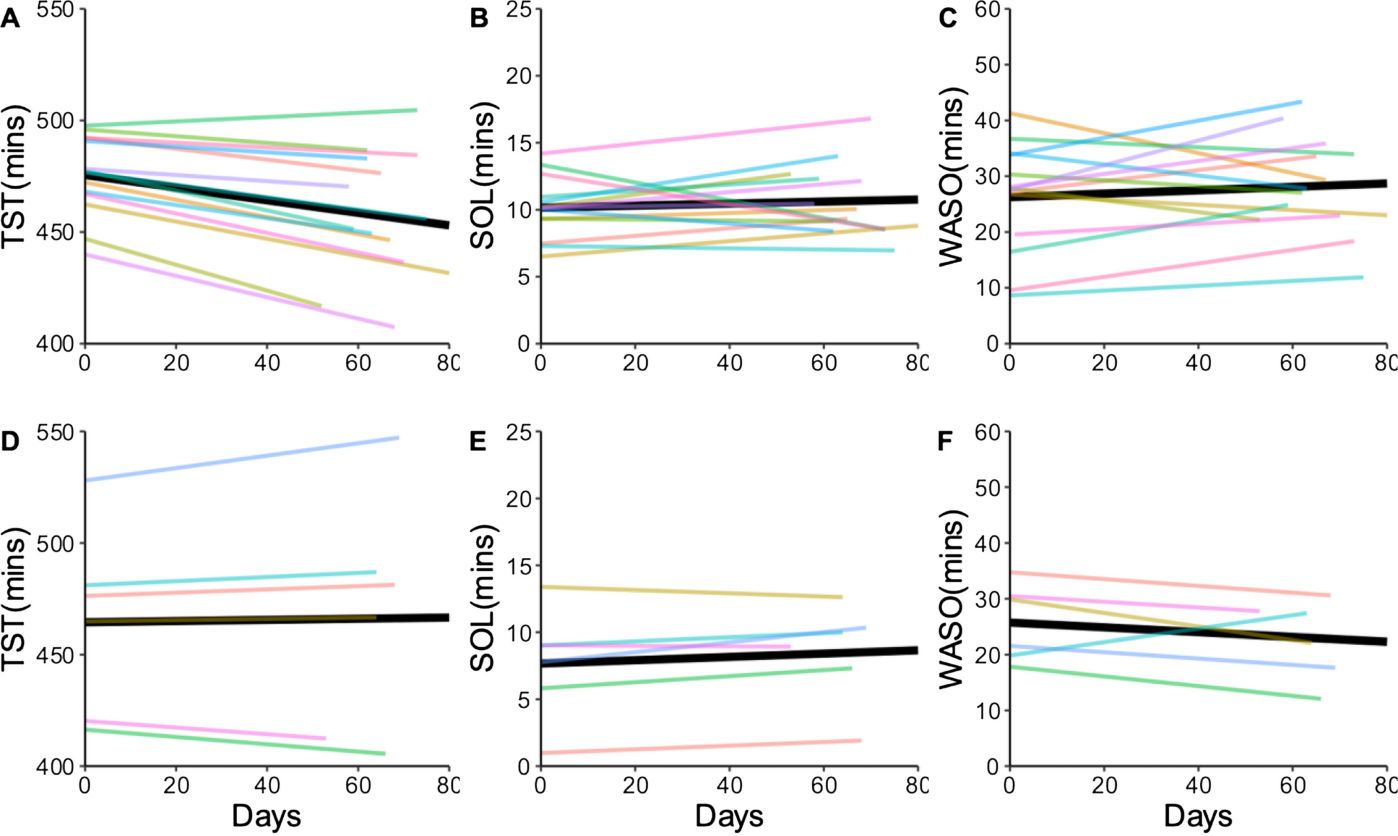
Linear mixed-effects model of sleep duration and quality. A, B, and C are the high group models; D, E, and F are the low group models.

Total sleep time intercepts were 475.54 minutes (*P* < .001; confidence interval [CI], 463.22–487.86 minutes [7.93 hours]) per night in the high engagement group and 464.47 minutes (*P* < .001; CI, 428.23-500.70 minutes [7.74 hours]) per night in the low engagement group. The TST slope values were −0.28 (*P* = .015; CI, −0.51 to 0.05) in the high engagement group and 0.03 (*P* = .889; CI, −0.35 to 0.40) in the low engagement group. Like the WASO comparisons, both groups started at similar TST baseline values, but the low group experienced an improvement in TST over time, whereas the high group showed a decrease in TST over time (Figure 2).

**FIGURE 3:**
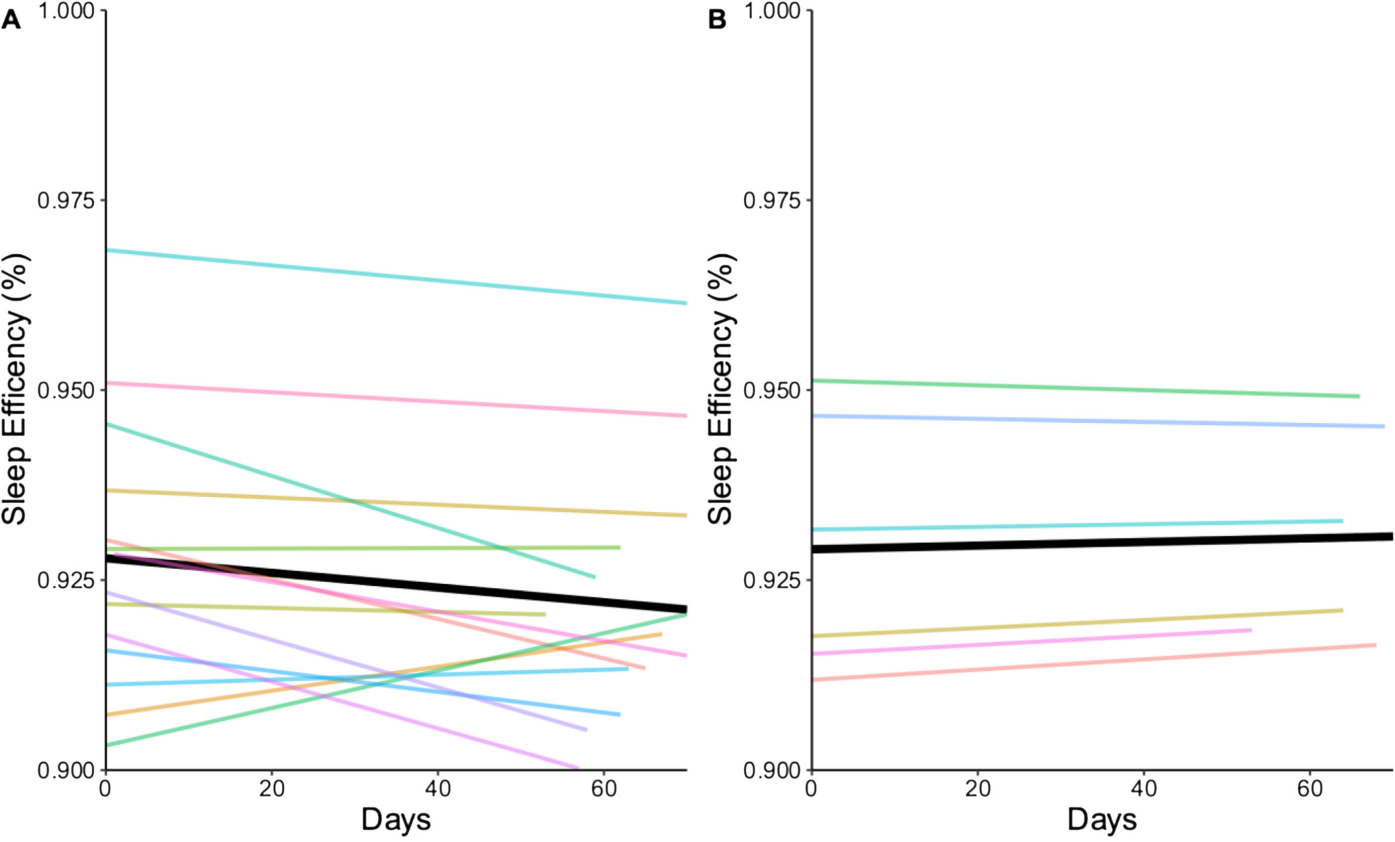
Linear mixed-effects model of sleep efficiency. A = high group model; B = low group model.

The SOL intercept for the high engagement group was 11.16 minutes (*P* < .001; CI, 8.79–13.52 minutes) and 8.33 minutes (*P* < .001; CI, 4.20–12.47 minutes) for the low use group. The SOL slope values were similar, 0.02 in the low group (*P* = .523; CI, −0.03 to 0.07) and 0.03 (*P* = .293; CI, 0.02–0.08) in high group. The groups had different baseline SOL times, and the low engagement group experienced a slight increase, trending toward values above 5 minutes. The WASO intercept in the high engagement group was 26.26 minutes (*P* < .001; CI, 20.36–32.17 minutes) and 25.73 minutes (*P* < .001; CI, 19.06–32.41 minutes) in the low engagement group. The WASO slope values were 0.03 (*P* = .554; CI, −0.07 to 0.13) in the high engagement group and −0.04 (*P* = .492; CI, −0.17 to 0.08) in the low engagement group; the groups began at a similar baseline, and the low engagement group experienced a slight decrease in WASO over time. The sleep efficiency intercepts in the high and low user groups were 93% (high: *P* < .001; CI, 0.92–0.94, low: *P* < .001; CI, 0.91–0.94). The groups started at the same sleep efficacy percentage at the start of the pilot, and the low user group trended toward increased sleep efficiency, whereas the high user group trended toward a decrease in sleep efficiency (Figure 3).

### Stress Levels Changes Over Time According to Engagement Group

**FIGURE 4:**
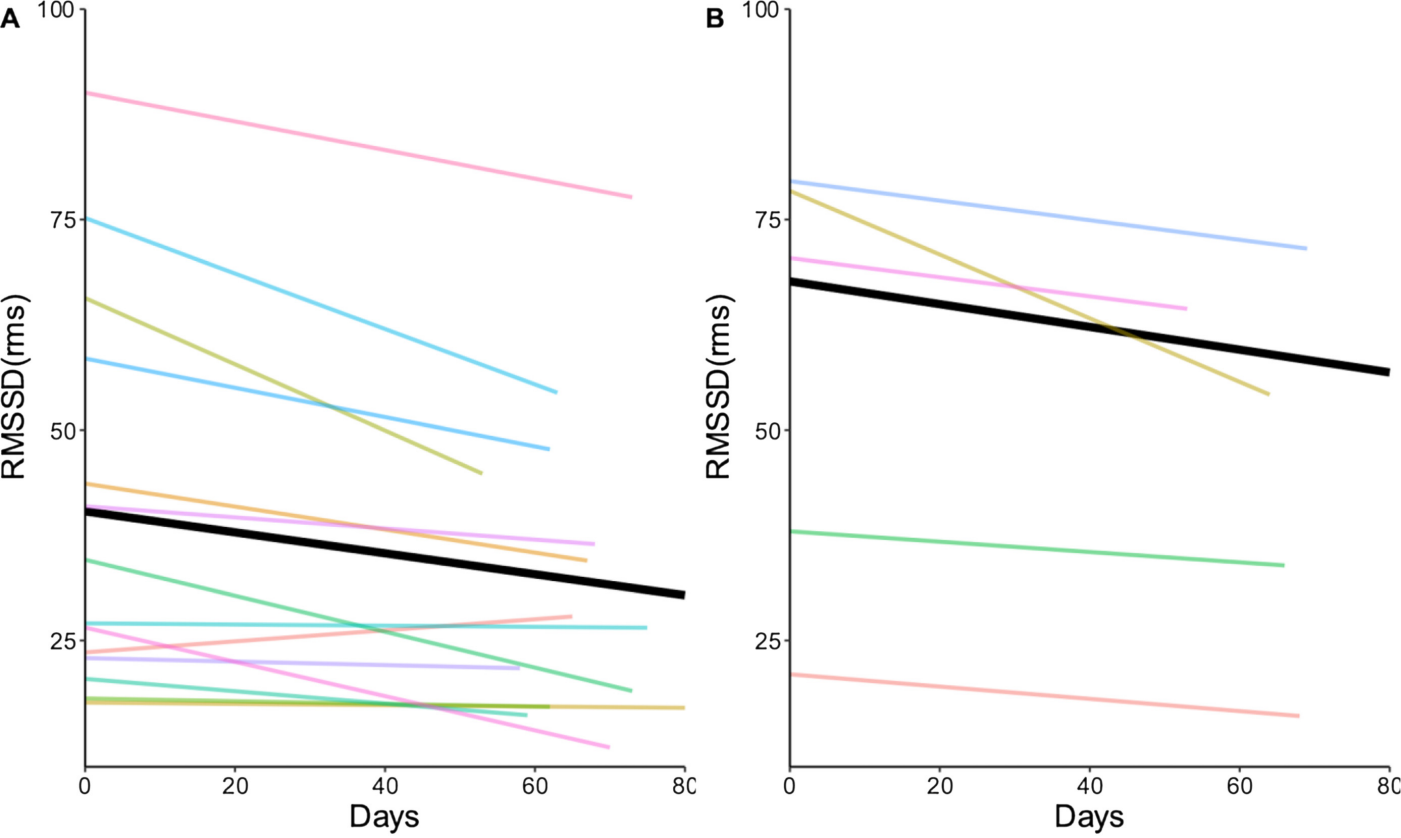
Linear mixed-effects model of RMSSD. A = high group model; B = low group model. Abbreviation: rms, root mean square.

The intercepts of the RMSSD were 40.35 (*P* < .001; CI, 28.09–52.60) in the high engagement group and 67.69 (*P* < .001; CI, 40.01–95.36) in the low group. The RMSSD slope values were −0.12 (*P* = .001; CI, −0.20 to −0.05) in the high engagement group and −0.14 (*P* = .023; CI, −0.25 to 0.02) in the low engagement group. Both groups experienced a decrease in RMSSD, an indication of normal changes over the course of pregnancy; however, the high engagement group experienced a lower value of RMSSD from the start of the study than did the low user group (Figure 4).

## DISCUSSION

### Main Findings

The study findings reveal that 70% of the women in our study were highly engaged in wearing the smart ring on their fingers for the duration of the pilot. Trends for sleep duration and quality were less favorable in the high engagement group than in the low engagement group. The high engagement group experienced a greater impact on their parasympathetic nervous system from stress exposure than did the low engagement group. Both groups had positive trends in SOL.

The demand for wearing a smart ring that monitors sleep and stress data was high in our pilot user group. Most users (80%; n = 16) missed less than 15% of data recording during the night, resulting a low level of missingness in our data set (Supplemental Digital Content 2, http://links.lww.com/CIN/A202). In our study, participants in the low engagement group experienced a barrier to wear the ring due to restrictions at work (not being able to wear rings during working hours). The demand for using eHealth programs has been shown in other pilot studies evaluating eHealth technologies. In Lima, Peru, researchers saw a similar percentage of physical use of their self-monitoring program for sleep and physical activity in a group of 20 women (65%; n = 13).^7^ The demand for eHealth integration into perinatal care has been noted in the contexts of labor and early discharge of infants and mothers from hospital.^29,40^

Second trimester is the period in which the sleep duration and quality are generally improved from the first trimester and generally worsen as the trimester ends.^4^ Our study findings reveal expected trends for both TST and WASO during the second trimester. We noted that the high engagement group trended toward the normal decline in TST as the second trimester progressed, and the low engagement group maintained a consistent duration of sleep throughout the pilot phase. A greater proportion of women in the high engagement group (64.28%) had one or more children than in the low engagement group (50.00%). Perhaps the group with more children will have experienced increased daily work related to childcare and perhaps experienced disrupted sleep due to needing to care for small children in the nighttime periods.

The women in the high engagement group also experienced higher impact from stress on their parasympathetic nervous system during the pilot period than did the women in the low engagement group. Factors related to daily patterns of living (eg, physical activity, amount of time at work, and life stresses) could have influenced the groups differently. Perhaps the group who performed more self-monitoring with the smart ring device experienced stress from the responsibility of knowing that they were recording correctly and not forgetting to wear and charge the ring effectively. The concern that burdening healthcare users with greater responsibilities in respect to their care could impact poorly on the users' levels of stress due to feelings of needing to perform, low health and eHealth literacy levels, and incompatibility between the digital service and the preferences of the individual.^16,17,19^ Self-monitoring users living with multiple sclerosis found that an important component of a technological self-monitoring program would be to have expert coaching and support for using this service related to the practical matters of self-monitoring technology and that the data collected should be integrated into the development of personalized treatment plans.^17^

### Implications for Future Research

Women in the high engagement group experienced higher sleep quality levels than did the low user group, based on SOL trends. Causes of sleep disturbances are varied, and for pregnant women in our study, their lives were impacted by a global pandemic with first lockdown orders starting on March 12, 2020. Another cohort of pregnant Finnish women participated in a cohort study examining sleep and physical activity patterns during the pandemic lockdown, and it was noted that the coping capacity of these women to maintain appropriate levels of stress and restful sleep habits (waking up later in the morning) might have been connected to a change in lifestyle habits due to the lockdown measures and the strong social supports available to Finnish nationals.^12^ Further research should focus on testing technological self-monitoring of sleep and stress in pregnancy with other population groups who experience different socioeconomic circumstances, diverse life experiences, and varying levels of behavioral engagement in the technological programs.

Our study demonstrates the usefulness of collecting large data sets from a valid home monitoring device. Although other studies have used valid sleep parameter data to understand sleep disturbances in pregnancy, these studies either have relied on short-term data collection in clinical settings or have used actigraphy monitoring paired with self-report of sleep.^24,25^ One recent pilot randomized controlled trial used a Shine 2 device to monitor sleep patterns at home for 12 weeks in 12 pregnant women randomized to sleep education and digital self-monitoring compared with a group of 12 pregnant women who only received sleep education.^30^ This study showed no significant differences between the groups on sleep questionnaire results; however, the study did not report on sleep parameter data measured with the Shine 2 device. Patient-reported outcome measures have their limitations and benefit from being paired with real-time sleep parameter recordings to understand validity of testing, which our study was able to provide.

The validation of the ŌURA sleep monitoring was explained to participants in our study. Women used the wearable device at high or low levels. Women's moderate to high willingness to use the smart ring to monitor sleep was dependent on how they felt about the trustworthiness of wearable data being collected during our study. Other studies have used sleep monitoring devices but have not reported the level of wear time throughout the study periods.^25,30^ By implementing this pilot study with less controls on when and for how long women would wear the smart ring, we could examine how much women would choose to or be able to use the smart device. Studies completed about engagement in self-monitoring have been conducted using smart bands and have studied physical activity and prevention of gestational weight gain.^30,41^ As well, because some of these studies implemented pay incentives to use the wearable device, this may have impacted participants' willingness to use the devices^41^; we provided no financial incentives to participants in our study. Our participants had free use of the device and mobile app and access to their data during the study period, which might have an impact on their desire to use the service on a trial basis.

The behavioral engagement examined in this pilot study was not consistently associated with positive sleep and stress outcomes. One reason for this could be related to the fact that optimal use of devices is not best thought of as a linear progression to higher and higher use, as some healthcare theorists have suggested.^19,42^ Rather, personalization and giving choice of how and when to use eHealth programs have been thought to lead to better outcomes and patient satisfaction in a perinatal care context.^2,43,44^ However, current research has yet to test the association of personalized eHealth programs in pregnancy and health outcomes directly. This an area of research that should be undertaken in the future.

### Implications for Nursing Practice

Tailoring the care processes toward individual pregnant users is critical for the practicing of woman-centered care. It is possible that the promise of access to data will lead to higher levels of digital engagement; however, emotional responses to seeing the trends of one's personal lifestyle habits might influence feelings of shame for underperforming or undue worry about the state of their unborn child in a case that they do not practice healthy enough habits.^45,46^ Each perinatal client can be guided to use the eHealth programs to the optimal level that suits their needs, preferences, and capacity to manage their own health promotional care.

Many studies have compared digital self-monitoring with regular health promotion interventions in pregnancy and concluded no significant difference between user groups' behavioral change activities and health outcomes.^30,31^ Health anthropologist Annemarie Mol states in *The Logic of Care* that “What characterizes good care is a calm, persistent but forgiving effort to improve the situation of a [client] or to keep this [condition] from deteriorating.”^19^ In light of what good care might be defined as care providers and eHealth developers should consider, low and high engagement in eHealth programs might lead to positive outcomes as long as the care process includes collaboration with perinatal care providers.

## LIMITATIONS

This study includes a small sample size; however, the numbers of observations we incorporated into the statistical analysis were high in volume. This is not a controlled study; the results are not generalizable, and more controlled interventions would be necessary to design any effectiveness studies. The descriptive comparative findings of this study highlight the need to better define the concept of behavioral engagement and to challenge our assumptions regarding the impact behavioral engagement has in the context of technological perinatal care processes. Our study is limited in the potential to see impacts on outcomes related to behavioral engagement as the women used the service during the second trimester and there are generally fewer disruptions to sleep during this period. We did observe women at a time when they were likely to experience sleep disruptions and high levels of stress due to other factors such as childrearing of their older children and due to the timing of the pilot, during a lockdown period related to the global SARS-CoV-2 pandemic.

## CONCLUSION

The use of self-monitoring technology allowed pregnant users, public health nurses, and health researchers to view and store sleep (duration and quality) and stress data in real time. Women in the high engagement group did not experience an improvement in sleep duration or quality compared with the women in the low engagement group, whereas women in the low group did experience higher scores in RMSSD and saw a less dramatic drop in their RMSSD value, an indication of less stress response. These findings may explain that personalization of self-monitoring strategies and meaningful, trusting interactions with health coaches play equally important roles in supporting pregnant persons and women toward health promotion activities as do initiatives to support increased behavioral engagement in self-monitoring.

## Supplementary Material
