# Supplementary material for: A Descriptive Comparative Pilot Study: Association Between Use of a Self-monitoring Device and Sleep and Stress Outcomes in Pregnancy
Source: Comput Inform Nurs. 2022 Nov 28;41(6):457–66. doi: 10.1097/CIN.0000000000000958 (PMC10241436; doi:10.1097/CIN.0000000000000958)
Supplement: Supplementary file 1 [file cin-41-457-s001.docx]

**Abstract**

Pregnancy is a challenging time for maintaining quality sleep and managing stress. Digital self-monitoring technologies are popular due to assumed increased patient engagement leading to an impact on health outcomes. However, the actual association between wear-time of such devices and improved sleep/stress outcomes remains untested. Here, a descriptive comparative pilot study of twenty pregnant women was conducted to examine associations between wear-time (behavioral engagement) of self-monitoring devices and sleep/stress pregnancy outcomes. Women used a ring fitted to their finger to monitor sleep/stress data, with access to a self-monitoring program for an average of 9 and a half weeks. Based on wear time, participants were split into two engagement groups. Using a linear mixed effect model, the high engagement group showed higher levels of stress and a negative trend in sleep duration, and quality. The low engagement group showed positive changes in sleep duration, and quality, and experienced below normal sleep onset latency at the start of the pilot but trended toward normal levels. Engagement according to device wear-time was not associated with improved outcomes. Further research should aim to understand how engagement with self-monitoring technologies impacts sleep/stress outcomes in pregnancy.

**Keywords:** Pregnancy; Wearable Sensors; Sleep; Self Care; Behavioral Changes

**Background**

Pregnancy is a time of physical and emotional changes. Many pregnant persons experience pain, discomfort, and bodily changes that have been linked to sleep disturbances which can increase the prenatal experience of stress.^1,2^ Sleep disturbances are common during pregnancy due to hormonal and physiological changes and manifest as insomnia or sleep fragmentation.^3,4^ Antenatal stress and sleep disturbances have been linked to increased likelihood of preterm birth. ^5,6^ Sleep disturbances have also been associated with incidence of still birth and growth and weight restrictions.^6^ Because of a low frequency of contact visits during the early antenatal period non-medical concerns such as sleep disturbances and stress in early pregnancy have been historically left out of antenatal care.^2^ It is now possible to monitor sleep quality and mental health states linked to stress during pregnancy using eHealth modalities.^7–10^ A self-monitoring technology that provides insights into actual sleep patterns and stress responses could support pregnant persons toward building a greater bodily awareness in between antenatal clinic visits.^11,12^

If pregnant persons are given access to sleep and stress data collected using 3D accelerometer, gyroscope and biomarker signals including Photoplethysmogram (PPG) and Electrocardiogram (ECG) through wearable recording devices and Bluetooth technology, they might be able to engage in lifestyle self-monitoring that stimulates their commitment to manage their sleep disturbances and levels of stress. Increasing pregnant persons’ commitment (behavioral engagement)^13^ over time might influence the quality of care.^14^

Pregnant persons use eHealth modalities to remind them about important issues during pregnancy when so much is already on their minds.^15^ Self-monitoring technology is being used by persons living with chronic conditions such as diabetes and multiple sclerosis in order to motivate and support behavioral engagement in self-care.^16,17^ These eHealth users experienced greater condition awareness, and benefited from setting goals toward health behaviors.^16,17^ The use of self-monitoring technologies has raised some concerns about user over-burden due to feelings of needing to perform after viewing their personal data, and experiences of decision fatigue related to access and choice of self-monitoring devices.^17–19^ Participation in self-care could be enhanced with the use of wearable devices and viewing of personal data however associations between behavioral engagement and health outcomes is not well understood.

It is common for pregnant persons to experience sleep disturbances during pregnancy, and the effects of such disruptions to normal rest and sleep have been shown to be positively correlated with fatigue, childbirth fear and anxiety.^20^ Although sleep and stress are important clinical problems, we know that personalized antenatal health promotion coaching for sleep and stress is often difficult due to the common inaccuracy of visit self-reports of stress, mental health states, and sleep quality.^21,22^ This results in an assessment gap at the time of clinic visits. In the past decade self-monitoring of personal health data has made it possible to monitor sleep duration and quality, and the levels of stress during pregnancy in between visits.^12,23^ Although the assessment gap can be lessened with the use of these technologies, little is understood about the impact technological interventions of self-monitoring might have on behavioral engagement in self-care, and subsequent quality of care and health outcomes. Previous studies investigating perinatal technological sleep self-monitoring have not investigated the association between behavioral engagement and health outcomes using multiple week collection of PPG signal sleep and stress parameters.^24,25^

**Self-monitoring and the engaged user**

Technological self-monitoring care processes are often aimed at motivating users to become engaged in their own self-care. ^26–28^ Having access to wearable devices and personal sleep and stress data makes it possible for pregnant persons and health care providers to assess health without a greater use of health service resources related to in person clinic visits. ^2,29^ Although self-monitoring technology is gaining popularity in perinatal care in high to middle income countries the associations between behavioral engagement in wearing devices and stress and sleep outcomes remain unclear. Studies examining the effectiveness of self-monitoring in pregnancy have revealed conflicting results, more should be investigated on the impact of self-monitoring activities on improved health outcomes.^8,9,30,31^

Wearable device monitoring and viewing personal data has shown to be highly effective and reliable for use in daily life of users and for research purposes. ^11,23,32^ Self-monitoring modalities support the collection of data about stress levels, and sleep duration, and quality.^12,23^ Changes overtime can also be examined related to pregnant users’ behavioral engagement of wearing devices (e.g. wear-time). The objective of this pilot study was to observe any associations between pregnant persons’ behavioral engagement and changes in sleep duration, and quality and levels of stress. Behavioral engagement was measured using amount of wear time of the smart ring device (worn on the finger).

**Methods**

The pilot study investigated the implementation and demand of using a smart ring self-monitoring technology in a Finnish antenatal clinic.^33^ This study is one phase of a larger feasibility study examining engagement by pregnant persons in a perinatal eHealth program using self-monitoring and goal-setting for physical activity, stress and sleep in collaboration with their public health nurses. In the present pilot report implementation and demand were examined by comparing the user groups according to their level of behavioral engagement (i.e. wear-time) and their trends in sleep duration and quality and levels of stress over the course of the pilot period.^33^

**Study Participants and Setting**

Pregnant persons receiving care at one antenatal clinic in South-West Finland were sampled using convenience sampling.^34^ Participants were enrolled in the study between March and August 2020 during their first or early second trimesters. Inclusion criteria included being 18 years or older, having access to a smartphone (Android or iOS) and having good literacy in Finnish and English languages. Six public health nurses were enrolled in the larger feasibility study, they received the smart rings and use of the wellness web and smartphone Applications (Apps) at the start of the study to familiarize themselves with the use of the self-monitoring technology. Public health nurses agreed to participate in the larger feasibility study to act as testers and supports for the pregnant persons who used the self-monitoring and goal setting eHealth program. Participants were recruited during late first trimester of pregnancy or early to mid-second trimester to allow for a nurse visit schedule to include two or more visits prior to the end of the larger feasibility study.

**Pilot Use of ŌURA Technology**

Pregnant persons who consented to take part in the study received the wearable ring to be worn on their finger and access to the smartphone and web ŌURA Apps through Bluetooth pairing and anonymized user logins. The ŌURA technology is a commercially available wearable device to be worn on the finger. Version 2.0 was used in this study and was able to monitor sleep and stress data. The stress levels were interpreted from the recording of heart rate variability during sleep. All night-time recordings can be uploaded to the smartphone and web Apps through a Bluetooth connection each day. Users could access the web App to view more details on their data trends and download their own data if they wished. The smartphone App provided daily tips and feedback about best practices for maintaining low stress levels and sleep duration and quality. As a part of the larger feasibility study pregnant participants were instructed to wear the ring as much as possible every day in ways that best suited them. They were instructed also to discuss use of the smart ring and Apps, self-monitoring, and goal setting for physical activity, stress, and sleep with their public health nurse over the course of the study.

**Data Collection**

Participants piloted the self-monitoring program for on average 9.5 weeks. The smart ring device has been tested and validated for the monitoring of sleep and heartrate variability data. ^11,32,35^ Participants in our study recorded daily sleep, and stress data whenever they wore the smart ring. Participants completed demographic, use and availability of technology, and health parameter survey data at baseline of the study period. Participants were informed that if the smart ring was uncomfortable, not recording well, or the battery did not last between normal charging periods to contact the nurse researcher for assistance. The data were uploaded with Bluetooth pairing to the ŌURA cloud service supported by a data sharing and storing system provided through the ŌURA company.

**Non-wear time**

A record is kept through the PPG signal detection of the smart ring indicating when the ring is being worn. Total minutes of non-wear time per day was recorded by the device and uploaded to the cloud storage through Bluetooth connection.

**Sleep duration and quality**

Total sleep time (TST) is a measure of duration of total sleep during the night.^4^ Sleep quality was measured in our study using sleep onset latency (SOL), wake after sleep onset (WASO), and sleep efficiency. SOL is the time it takes to move from a fully wakeful state to a sleep state determined by Polysomnography.^36^ The ŌURA ring 2.0 has been validated to measure this parameter using PPG signal and hand movement indicators (e.g., accelerometer).^11^ SOL is commonly experienced as equal to or less than 20 minutes.^36^ WASO was recorded in the length of time spent awake after sleep onset, this indicated how much sleep disturbance is experienced according to disrupted total sleep time.^4^ Sleep efficiency was calculated by dividing TST by the sum of TST, SOL, and WASO.

**Levels of stress**

The root mean square of successive differences (RMSSD) reflects the variance in heartrate beat-to-beat and is a primary time-domain measure for estimating the vagally mediated changes reflected in heartrate variability.^37^ Lower values of RMSSD are indicative of increased impact on the parasympathetic nervous system as a response to physiological stress exposure.^38^

**Statistical analysis**

**Data Pre-processing**

ŌURA smart ring provides the daily data summary for sleep and stress parameters in a structured format. We utilized Python 3.8 to parse these files and extract parameters we were interested in. Since ŌURA reports all the sleep events, we labelled the ones happening during night-time and focused only on night sleep.

Descriptive statistics (means, ranges, and distribution of values) of participants demographic and questionnaire totals were organized and prepared for analysis using R for statistical analyses (Version 3.6.1).

**Kernel Density Estimate analysis**

To cluster our subjects into high and low engagement groups, we extracted the non-wear time of the smart ring and looked at the normalized distributions and observed two groups of users based on the characteristics of the distributions. We leveraged Kernel Density Estimation (KDE) on the non-wear time to estimate such normalized distributions. KDE is a useful non-parametric tool to estimate the distributions and helps to distinguish different clusters of data.

**Linear mixed effect model analysis**

To model the characteristics of the participants, a hierarchical linear mixed model was exploited for each of the high and low engagement groups (see Supplemental Digital Content 1, http://links.lww.com/CIN/A201). Using this model, we were able to analyze the between-subject, within-subject, as well as overall trends. The single within-subject independent variable was the time (day) and the health outcomes related to sleep duration and quality and levels of stress were the dependent variables in this study.

**Ethics**

Ethical approval was obtained by the Ethics Committee of the Hospital District of Southwest Finland prior to the start of the study (Approval ID: ETMK Dnro: 1/1801/2020). Pregnant persons and public health nurses provided informed consent before participation in the larger feasibility study.

**Findings**

Six public health nurses and 20 pregnant women agreed to participate in the larger feasibility study. Finnish pregnant women joined the study during their first or early second trimesters. All women had low risk pregnancies at the start of the study with one participant requiring bed rest later in the study period. Eighteen (90%) of the women were employed, and seven (35%) of the women experienced chronic illnesses outside of pregnancy.

Seventy percent of women in the study stated that their pregnancies negatively impacted their sleep quality (71.4%; n=10 of high engagement group; 66.6%; n=4 of low engagement group). All participants stated that they had an unlimited smartphone data plan to use in the study. We experienced some technical difficulties regarding appropriate smart ring sizes and faulty batteries, however women received fast technical service and new smart rings within 24 hours of their reported concerns (see Supplemental Digital Content 2, http://links.lww.com/CIN/A202).

**Engagement measured by wear time: Kernel Density Estimate (KDE) Analysis**

Women were spilt into high and low engagement group by clustering subjects with a normalized non-wear time less than 20% as high engagement group (n=14; 70%) and the rest as low engagement group. Distributions according to participants can be seen in Figure 1. See Table 1 for background data according to user groups.

[Insert Figure 1 here]

[Insert Table 1 here]

**Sleep duration, and quality changes over time according to engagement groups**

Total sleep time (TST) intercepts were 475.54 (P<.001; CI: 463.22-487.86) minutes (7.93 hours) per night in the high engagement group and 464.47 (P<.001; CI: 428.23-500.70) minutes (7.74 hours) per night in the low engagement group. TST slope values were -0.28 (*P*=.015; CI: -0.51-0.05) for the high engagement group and 0.03 (*P*=.889; CI: -0.35-0.40) in the low engagement group. Like the WASO comparisons both groups started at similar TST baseline values, but the low group experienced an improvement in TST over time whereas the high group showed a decrease in TST over time.

The Sleep Onset Latency (SOL) intercept for the high engagement group was 11.16 minutes (P <.001; CI: 8.79-13.52) and 8.33 minutes (P<.001; CI: 4.20-12.47) for the low use group. SOL slope values were similar, 0.02 in the low group (*P*=.523; CI: -0.03-0.07) and 0.03 (*P*=.293; CI: 0.02-0.08) in high group. The groups had different baseline SOL times and the low engagement group experienced a slight increase, trending toward values above 5 minutes. Waking after sleep onset (WASO) intercept in the high engagement group was 26.26 minutes (P<.001; CI: 20.36-32.17) and 25.73 minutes (P<.001; CI: 19.06-32.41) in the low engagement group. WASO slope values were 0.03 (*P*=.554; CI: -0.07-0.13) in the high engagement group and -0.04 (*P*=.492; CI: -0.17-0.08) for the low engagement group, the groups began at a similar baseline and the low engagement group experienced a slight decrease in WASO over time. The sleep efficiency intercept in the high and low user groups were 93% (high: P<.001; CI: 0.92-0.94, low: P<.001; CI: 0.91-0.94). The groups started at the same sleep efficacy percentage at the start of the pilot and the low user group trended toward increased sleep efficiency whereas the high user group trended toward a decrease in sleep efficiency (See Figure 3).

[Insert Figure 2 here]

[Insert Figure 3 here]

**Stress levels changes over time according to engagement group**

The intercepts of the Root Mean Square of successive differences (RMSSD) were 40.35 (P<.001; CI: 28.09-52.60) in the high engagement group and 67.69 (P<.001; CI: 40.01-95.36) in the low group. RMSSD slope value of the high engagement group was -0.12 (*P*=.001; CI: -0.20- -0.05) and -0.14 (P=.023; CI: -0.25-0.02) in the low engagement group. Both groups experienced a decrease in RMSSD, an indication of normal changes over the course of pregnancy, however the high engagement group experienced a lower value of RMSSD from the start of the study than did the low user group.

[Insert Figure 4 here]

**Discussion**

**Main Findings**

The study findings reveal that 70% of the women in our study were highly engaged in wearing the smart ring on their fingers for the duration of the pilot. Trends for sleep duration and quality were less favorable in the high engagement group than in the low engagement group. The high engagement group experienced a greater impact on their parasympathetic nervous system from stress exposure than did the low engagement group. Both groups had positive trends in SOL.

The demand for wearing a smart ring that monitors sleep and stress data was high in our pilot user group. Most users (80%; n=16) missed less than 15% of data recording during the night, resulting a low level of missingness in our data set (see Supplemental Digital Content 2, http://links.lww.com/CIN/A202). In our study participants in the low engagement group experienced a barrier to wear the ring due to restrictions at work (not being able to wear rings during working hours). The demand for using eHealth programs has been shown in other pilot studies evaluating eHealth technologies. In Lima, Peru researchers saw a similar percentage of physical use of their self-monitoring program for sleep and physical activity in a group of 20 women (65%; n=13).^7^ The demand for eHealth integration into perinatal care has been noted in the contexts of labor and early discharge of infants and mothers from hospital.^29,39^

Second trimester is the period in which the sleep duration and quality are generally improved from the first trimester and generally worsen as the trimester ends.^4^

Our study findings reveal expected trends for both TST and WASO during the second trimester. We noted that the high engaged group trended toward the normal decline in TST as the second trimester progressed and the low engagement group maintained a consistent duration of sleep throughout the pilot phase. A greater proportion of women in the high engagement group (64.28%) had 1 or more children than in the low engagement group (50.00%). Perhaps the group with more children will have experienced increased daily work related to childcare and perhaps experience disrupted sleep due to needing to care for small children in the night-time periods.

The women in the high engagement group also experienced higher impact from stress on their parasympathetic nervous system during the pilot period than the women in the low engagement group. Factors related to daily patterns of living (e.g. physical activity, amount of time at work and life stresses) could have influenced the groups differently. Perhaps the group that performed more self-monitoring with the smart ring device experienced stress from the responsibility of knowing that they were recording correctly and not forgetting to wear and charge the ring effectively. The concern that burdening healthcare users with greater responsibilities in respect to their care could impact poorly on the users’ levels of stress due to feelings of needing to perform, low health and eHealth literacy levels, and incompatibility between the digital service and the preferences of the individual.^16,17,19^ Self-monitoring users living with multiple sclerosis found that an important component of a technological self-monitoring program would be to have expert coaching and support for using this service related to the practical matters of self-monitoring technology and that the data collected should be integrated into the development of personalized treatment plans.^17^

**Implications for Future Research**

Women in the high engagement group experienced higher sleep quality levels than the low user group, based on SOL trends. Causes of sleep disturbances are varied and for pregnant women in our study their lives were impacted by a global pandemic with first lockdown orders starting on March 12, 2020. Another cohort of pregnant Finnish women participated in a cohort study examining sleep and physical activity patterns during the pandemic lockdown and it was noted that the coping capacity of these women to maintain appropriate levels of stress and restful sleep habits (waking up later in the morning) might have been connected to a change in lifestyle habits due to the lockdown measures and the strong social supports available to Finnish nationals.^12^ Further research should focus on testing technological self-monitoring of sleep and stress in pregnancy with other population groups who experience different socioeconomical circumstances, diverse life experiences, and varying levels of behavioral engagement in the technological programs.

Our study demonstrates the usefulness of collecting large data sets from a valid home monitoring device. While other studies have used valid sleep parameter data to understand sleep disturbances in pregnancy these studies have relied on either short-term data collection in clinical settings or have used actigraphy monitoring paired with self-report of sleep.^24,25^ One recent pilot randomized controlled trial used a Shine 2 device to monitor sleep patterns at home for 12 weeks in 12 pregnant women randomized to sleep education and digital self-monitoring compared to a group of 12 pregnant women who only received sleep education.^30^ This study showed no significant differences between the groups on sleep questionnaire results however the study did not report on sleep parameter data measured with the Shine 2 device. Patient reported outcome measures have their limitations and benefit from being paired with real-time sleep parameter recordings to understand validity of testing, which our study was able to provide.

The validation of the ŌURA sleep monitoring was explained to participants in our study. Women used the wearable device at high or low levels. Women’s moderate to high willingness to use the smart ring to monitor sleep was dependent on how they felt about the trustworthiness of wearable data being collected during our study. Other studies have used sleep monitoring devices but have not reported the level of wear time throughout the study periods.^25,30^ By implementing this pilot study with less controls on when and for how long women would wear the smart ring we could examine how much women would choose to or be able to use the smart device. Studies completed about engagement in self-monitoring have been conducted using smart bands, and have studied physical activity and prevention of gestational weight gain.^30,40^ As well some of these studies implemented pay incentives to use the wearable device, this may have impacted participants’ willingness to use the devices,^40^ we provided no financial incentives to participants in our study. Our participants had free use of the device and mobile application, and access to their data during the study period, which might have impact on their desire to use the service on a trial basis.

The behavioral engagement examined in this pilot study was not consistently associated with positive sleep and stress outcomes. One reason for this could be related to the fact that optimal use of devices is not best thought of as a linear progression to higher and higher use, as some healthcare theorists have suggested.^19,41^ Rather, personalization and giving choice of how and when to use eHealth programs has been thought to lead to better outcomes and patient satisfaction in a perinatal care context.^2,42,43^ However, current research has yet to test the association of personalized eHealth programs in pregnancy and health outcomes directly. This an area of research that should be undertaken in the future.

**Implications for Nursing Practice**

Tailoring the care processes toward individual pregnant users is critical for the practicing of woman-centered care. It is possible that the promise of access to data will lead to higher levels of digital engagement, however, emotional responses to seeing the trends of one’s personal lifestyle habits might influence feelings of shame for underperforming or undue worry about the state of their unborn child in a case that they do not practice healthy enough habits.^44,45^ Each perinatal client can be guided to use the eHealth programs to the optimal level that suites their needs, preferences, and capacity to manage their own health promotional care.

Many studies have compared digital self-monitoring to regular health promotion interventions in pregnancy and concluded no significant difference between user groups’ behavioral change activities and health outcomes.^30,31^ Health anthropologist Annemarie Mol states in the text Logic of Care, that “What characterizes good care is a calm, persistent but forgiving effort to improve the situation of a [client] or to keep this [condition] from deteriorating”.^19^ In light of what good care might be defined as care providers and eHealth developers should consider that low and high engagement in eHealth programs might lead to positive outcomes as long as the care process includes collaboration with perinatal care providers.

**Limitations**

This study includes a small sample size, however, the number of observations we incorporated into the statistical analysis were high in volume. This is not a controlled study, and the results are not generalizable and more controlled interventions would be necessary to design any effectiveness studies. The descriptive comparative findings of this study highlight the need to better define the concept of behavioral engagement and to challenge our assumptions regarding the impact behavioral engagement has in the context of technological perinatal care processes. Our study is limited in the potential to see impacts on outcomes related to behavioral engagement as the women used the service during the second trimester and there are generally less disruptions to sleep during this period. We did observe women at a time when they were likely to experience sleep disruptions and high levels of stress due to other factors such as childrearing of their older children and due to the timing of the pilot, during a lockdown period related to the global SARS-CoV-2 pandemic.

**Conclusion**

The use of self-monitoring technology allowed pregnant users, public health nurses and health researchers to view and store sleep (duration and quality) and stress data in real-time. Women in the high engagement group did not experience an improvement in sleep duration or quality compared to the women in the low engagement group. Whereas women in the low group did experience higher scores in RMSSD and saw a less dramatic drop in their RMSSD value, an indication of less stress response. These findings may explain that personalization of self-monitoring strategies and meaningful, trusting interactions with health coaches play equally important roles in supporting pregnant persons and women toward health promotion activities as do initiatives to support increased behavioral engagement in self-monitoring.

**References**

1. Kamysheva E, Skouteris H, Wertheim EH, Paxton SJ, Milgrom J. A prospective investigation of the relationships among sleep quality, physical symptoms, and depressive symptoms during pregnancy. *Journal of Affective Disorders*. 2010;123(1-3):317-320. doi:10.1016/j.jad.2009.09.015

2. de Mooij MJM, Hodny RL, O’Neil DA, et al. OB Nest: Reimagining Low-Risk Prenatal Care. *Mayo Clinic Proceedings*. 2018;93(4):458-466. doi:10.1016/j.mayocp.2018.01.022

3. Sedov ID, Cameron EE, Madigan S, Tomfohr-Madsen LM. Sleep quality during pregnancy: A meta-analysis. *Sleep Medicine Reviews*. 2018;38:168-176. doi:10.1016/j.smrv.2017.06.005

4. Balserak B, Lee K. *Sleep and Sleep Disorders Associated with Pregnancy,” InPrinciples and Practice of Sleep Medicine*. Sixth. Elsevier; 2017.p.1572-1586

5. Staneva A, Bogossian F, Pritchard M, Wittkowski A. The effects of maternal depression, anxiety, and perceived stress during pregnancy on preterm birth: A systematic review. *Women and Birth*. 2015;28(3):179-193. doi:10.1016/j.wombi.2015.02.003

6. Warland J, Dorrian J, Morrison JL, O’Brien LM. Maternal sleep during pregnancy and poor fetal outcomes: A scoping review of the literature with meta-analysis. *Sleep Medicine Reviews*. 2018;41:197-219. doi:10.1016/j.smrv.2018.03.004

7. Galea JT, Ramos K, Coit J, et al. The Use of Wearable Technology to Objectively Measure Sleep Quality and Physical Activity Among Pregnant Women in Urban Lima, Peru: A Pilot Feasibility Study. *Matern Child Health J*. 2020;24(7):823-828. doi:10.1007/s10995-020-02931-5

8. Muuraiskangas S, Mattila E, Kyttala P, Koreasalo M, Lappalainen R. User Experiences of a Mobile Mental Well-Being Intervention Among Pregnant Women. In: Serino S, Matic A, Giakoumis D, Lopez G, Cipresso P, eds. *Pervasive Computing Paradigms for Mental Health (Mindcare 2015)*. Vol 604. ; 2016:140-149. doi:10.1007/978-3-319-32270-4_14

9. Hawkins M, Iradukunda F, Paterno M. Feasibility of a Sleep Self-Management Intervention in Pregnancy Using a Personalized Health Monitoring Device: Protocol for a Pilot Randomized Controlled Trial. *Jmir Research Protocols*. 2019;8(5):118-128. doi:10.2196/12455

10. Doherty K, Marcano-Belisario J, Cohn M, et al. Engagement with Mental Health Screening on Mobile Devices: Results from an Antenatal Feasibility Study. In: *Proceedings of the 2019 CHI Conference on Human Factors in Computing Systems*. ACM; 2019:1-15. doi:10.1145/3290605.3300416

11. Asgari Mehrabadi M, Azimi I, Sarhaddi F, et al. Sleep Tracking of a Commercially Available Smart Ring and Smartwatch Against Medical-Grade Actigraphy in Everyday Settings: Instrument Validation Study. *JMIR Mhealth Uhealth*. 2020;8(10):e20465. doi:10.2196/20465

12. Niela-Vilén H, Auxier J, Ekholm E, et al. Pregnant women’s daily patterns of well-being before and during the COVID-19 pandemic in Finland: Longitudinal monitoring through smartwatch technology. Ryckman KK, ed. *PLoS ONE*. 2021;16(2):e0246494. doi:10.1371/journal.pone.0246494

13. Higgins T, Larson E, Schnall R. Unraveling the meaning of patient engagement: A concept analysis. *Patient Education and Counseling*. 2017;100(1):30-36. doi:10.1016/j.pec.2016.09.002

14. Schwappach DLB. Review: Engaging Patients as Vigilant Partners in Safety: A Systematic Review. *Med Care Res Rev*. 2010;67(2):119-148. doi:10.1177/1077558709342254

15. Himes KP, Donovan H, Wang S, Weaver C, Grove JR, Facco FL. Healthy Beyond Pregnancy, a Web-Based Intervention to Improve Adherence to Postpartum Care: Randomized Controlled Feasibility Trial. *JMIR Hum Factors*. 2017;4(4):e26. doi:10.2196/humanfactors.7964

16. Rollo ME, Aguiar EJ, Williams RL, et al. eHealth technologies to support nutrition and physical activity behaviors in diabetes self-management. *DMSO*. 2016;Volume 9:381-390. doi:10.2147/DMSO.S95247

17. Wendrich K, van Oirschot P, Martens MB, Heerings M, Jongen PJ, Krabbenborg L. Toward Digital Self-monitoring of Multiple Sclerosis. *International Journal of MS Care*. 2019;21(6):282-291. doi:10.7224/1537-2073.2018-083

18. Vorderstrasse A, Lewinski A, Melkus GD, Johnson C. Social Support for Diabetes Self-Management via eHealth Interventions. *Curr Diab Rep*. 2016;16(7):56. doi:10.1007/s11892-016-0756-0

19. Mol, Annemarie. *The Logic of Care: Health and the Problem of Patient Choice.* Routledge: Taylor & Francis Group; 2018. p.19

20. Hall WA, Hauck YL, Carty EM, Hutton EK, Fenwick J, Stoll K. Childbirth Fear, Anxiety, Fatigue, and Sleep Deprivation in Pregnant Women. *Journal of Obstetric, Gynecologic & Neonatal Nursing*. 2009;38(5):567-576. doi:10.1111/j.1552-6909.2009.01054.x

21. Solhan MB, Trull TJ, Jahng S, Wood PK. Clinical assessment of affective instability: Comparing EMA indices, questionnaire reports, and retrospective recall. *Psychological Assessment*. 2009;21(3):425-436. doi:10.1037/a0016869

22. Herring SJ, Foster GD, Pien GW, et al. Do pregnant women accurately report sleep time? A comparison between self-reported and objective measures of sleep duration in pregnancy among a sample of urban mothers. *Sleep Breath*. 2013;17(4):1323-1327. doi:10.1007/s11325-013-0835-2

23. Sarhaddi F, Azimi I, Labbaf S, et al. Long-Term IoT-Based Maternal Monitoring: System Design and Evaluation. *Sensors*. 2021;21(7):2281. doi:10.3390/s21072281

24. McIntyre JPR, Ingham CM, Hutchinson BL, et al. A description of sleep behaviour in healthy late pregnancy, and the accuracy of self-reports. *BMC Pregnancy Childbirth*. 2016;16(1):115. doi:10.1186/s12884-016-0905-0

25. Tsai SY, Lin JW, Kuo LT, Thomas KA. Daily Sleep and Fatigue Characteristics in Nulliparous Women during the Third Trimester of Pregnancy. *Sleep*. 2012;35(2):257-262. doi:10.5665/sleep.1634

26. Willcox JC, Wilkinson SA, Lappas M, et al. A mobile health intervention promoting healthy gestational weight gain for women entering pregnancy at a high body mass index: the txt4two pilot randomised controlled trial. *Bjog-an International Journal of Obstetrics and Gynaecology*. 2017;124(11):1718-1728. doi:10.1111/1471-0528.14552

27. Butler Tobah YS, LeBlanc A, Branda ME, et al. Randomized comparison of a reduced-visit prenatal care model enhanced with remote monitoring. *American Journal of Obstetrics and Gynecology*. 2019;221(6):638.e1-638.e8. doi:10.1016/j.ajog.2019.06.034

28. Marko K, Ganju N, Krapf JM, et al. A Mobile Prenatal Care App to Reduce In-Person Visits: Prospective Controlled Trial. *Jmir Mhealth and Uhealth*. 2019;7(5):e10520. doi:10.2196/10520

29. O’Brien E, Rauf Z, Alfirevic Z, Lavender T. Women’s experiences of outpatient induction of labour with remote continuous monitoring. *Midwifery*. 2013;29(4):325-331. doi:10.1016/j.midw.2012.01.014

30. Hsiao WH, Paterno MT, Iradukunda F, Hawkins’ M. The Preliminary Efficacy of a Sleep Self-management Intervention Using a Personalized Health Monitoring Device during Pregnancy. *Behavioral Sleep Medicine*. 2021;19(6):705-716. doi:10.1080/15402002.2020.1851230

31. Hantsoo L, Criniti S, Khan A, et al. A Mobile Application for Monitoring and Management of Depressed Mood in a Vulnerable Pregnant Population. *Psychiatric Services*. 2018;69(1):104-107. doi:10.1176/appi.ps.201600582

32. Cao R, Azimi I, Sarhaddi F, et al. *Accuracy Assessment of Oura Ring Nocturnal Heart Rate and Heart Rate Variability in Comparison to Electrocardiography: A Comprehensive Analysis in Time and Frequency Domains (Preprint)*. Journal of Medical Internet Research; 2021. doi:10.2196/preprints.27487

33. Bowen DJ, Kreuter M, Spring B, et al. How We Design Feasibility Studies. *American Journal of Preventive Medicine*. 2009;36(5):452-457. doi:10.1016/j.amepre.2009.02.002

34. Etikan I. Comparison of Convenience Sampling and Purposive Sampling. *AJTAS*. 2016;5(1):1. doi:10.11648/j.ajtas.20160501.11

35. Kinnunen H, Rantanen A, Kenttä T, Koskimäki H. Feasible assessment of recovery and cardiovascular health: accuracy of nocturnal HR and HRV assessed via ring PPG in comparison to medical grade ECG. *Physiol Meas*. 2020;41(4):04NT01. doi:10.1088/1361-6579/ab840a

36. Walia, Harneet K, Mehra, Reena. *Practical Aspects of Actigraphy and Approaches in Clinical and Research Domains.* Vol 160. Handbook of Clinical Neurology; 2019. https://doi.org/10.1016/B978-0-444-64032-1.00024-2

37. Shaffer F, McCraty R, Zerr CL. A healthy heart is not a metronome: an integrative review of the heart’s anatomy and heart rate variability. *Front Psychol*. 2014;5. doi:10.3389/fpsyg.2014.01040

38. Kim HG, Cheon EJ, Bai DS, Lee YH, Koo BH. Stress and Heart Rate Variability: A Meta-Analysis and Review of the Literature. *Psychiatry Investigation*. 2018;15(3):235-245. doi:10.30773/pi.2017.08.17

39. Boe Danbjørg D, Wagner L, Clemensen J. Designing, Developing, and Testing an App for Parents Being Discharged Early Postnatally. *Journal for Nurse Practitioners*. 2014;10(10):794-802. doi:10.1016/j.nurpra.2014.07.023

40. Krukowski R, Johnson B, Kim H, Sen S, Homsi R. A Pragmatic Intervention Using Financial Incentives for Pregnancy Weight Management: Feasibility Randomized Controlled Trial. *JMIR Form Res*. 2021;5(12):e30578. doi:10.2196/30578

41. Oudshoorn N. *Telecare Technologies and the Transformation of Healthcare.* Springer; 2011.p. 68

42. Holm KG, Brodsgaard A, Zachariassen G, Smith AC, Clemensen J. Parent perspectives of neonatal tele-homecare: A qualitative study. *Journal of Telemedicine and Telecare*. 2019;25(4):221-229. doi:10.1177/1357633X18765059

43. Isetta V, Lopez-Agustina C, Lopez-Bernal E, et al. Cost-Effectiveness of a New Internet-Based Monitoring Tool for Neonatal Post-Discharge Home Care. *J Med Internet Res*. 2013;15(2). doi:10.2196/jmir.2361

44. Lupton D. The digitally engaged patient: Self-monitoring and self-care in the digital health era. *Social Theory & Health*. 2013;11(3):256-270. doi:10.1057/sth.2013.10

45. Salmela T, Valtonen A, Lupton D. The Affective Circle of Harassment and Enchantment: Reflections on the ŌURA Ring as an Intimate Research Device. *Qualitative Inquiry*. 2019;25(3):260-270. doi:10.1177/1077800418801376

**Figure 1.** Values represent normalized values of non-wear time

**Table 1. ***Women were unable to wear the ring on their fingers for health and safety reasons during working hours**;****Medical conditions included: migraines; asthma; hypothyroidism; ulcerative colitis; & endometriosis

**Figure 2.** A, B, and C are the High group models; D, E, F are the Low group models

**Figure 3.** A=High group model; B=Low group models

**Figure 4.** rms=Root Mean Squares; A=High group model; B=Low group models
