# Supplementary material for: A Descriptive Comparative Pilot Study: Association Between Use of a Self-monitoring Device and Sleep and Stress Outcomes in Pregnancy
Source: Comput Inform Nurs. 2022 Nov 28;41(6):457–66. doi: 10.1097/CIN.0000000000000958 (PMC10241436; doi:10.1097/CIN.0000000000000958)
Supplement: Supplementary file 2 [file cin-41-457-s002.docx]

**Supplement Material**

[S1: Hierarchical Linear Mixed Model (HLMM) Clusters According to User Group 2](#_Toc101434937)

[Figure S1: HLMM Cluster Plot SOL 2](#_Toc101434938)

[Figure S2: HLMM Cluster Plot WASO 3](#_Toc101434939)

[Figure S3: HLMM Cluster Plot TST 4](#_Toc101434940)

[Figure S4: HLMM Cluster Plot Sleep Efficiency 5](#_Toc101434941)

[Figure S5: HLMM Cluster Plot RMSSD 6](#_Toc101434942)

[S2: Missing data description 7](#_Toc101434943)

[Table S1: Percentage of Missing data (n=20) 7](#_Toc101434944)

## S1: Hierarchical Linear Mixed Model (HLMM) Clusters According to User Group

### Figure S1: HLMM Cluster Plot SOL

### Figure S2: HLMM Cluster Plot WASO

### Figure S3: HLMM Cluster Plot TST

###
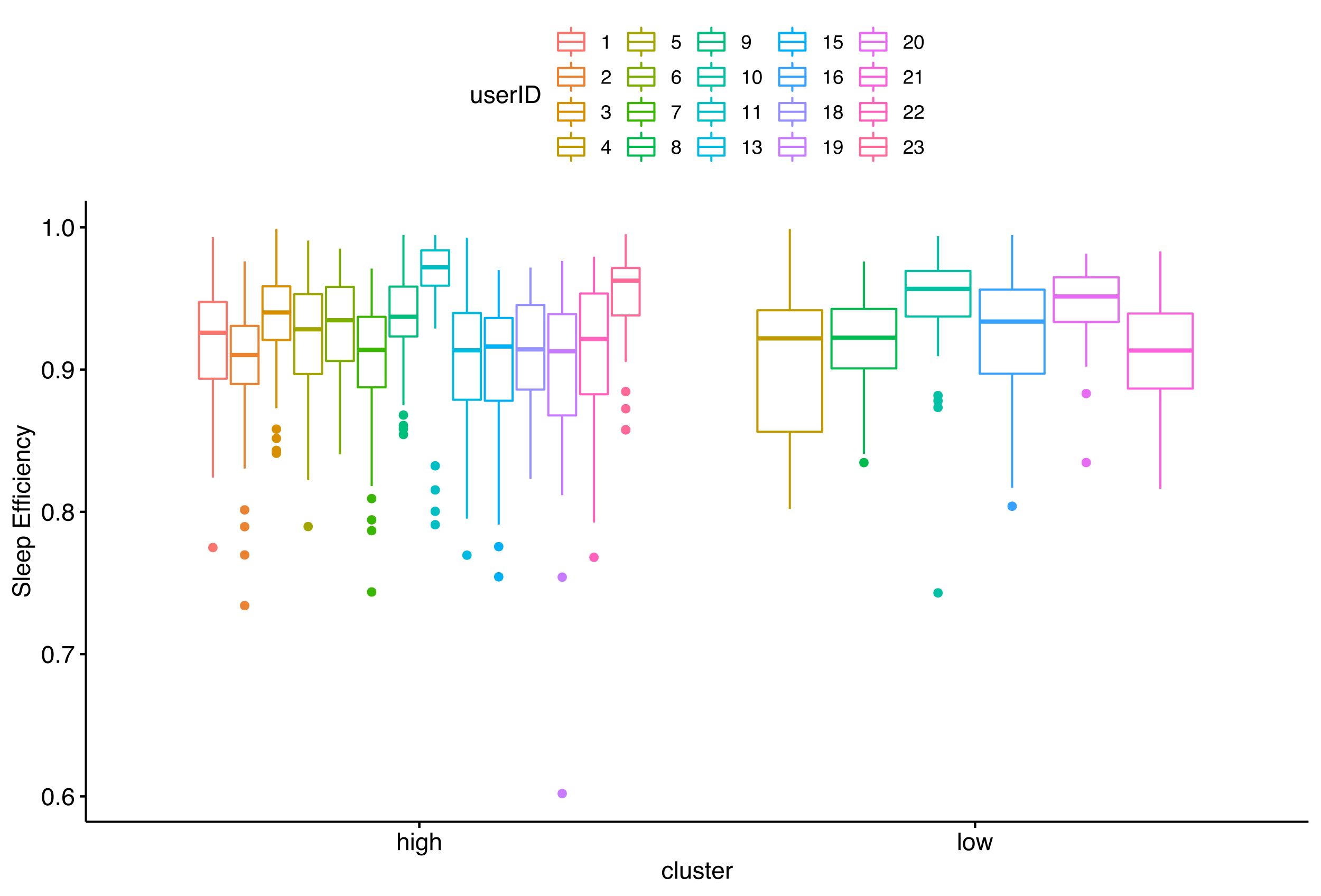
Figure S4: HLMM Cluster Plot Sleep Efficiency

### Figure S5: HLMM Cluster Plot RMSSD

## S2: Missing data description

### Table S1: Percentage of Missing data (n=20)

| ID | Sleep and Heart Parameters* (missing percentage) |
| --- | --- |
| 1 | 3.23 |
| 2 | 7.25 |
| 3 | 13.41 |
| 4 | 40.58 |
| 5 | 38.54 |
| 6 | 11.94 |
| 7 | 18.92 |
| 8 | 3.08 |
| 9 | 3.33 |
| 10 | 1.49 |
| 11 | 9.21** |
| 13 | 4.69 |
| 15 | 9.52 |
| 16 | 27.69 |
| 18 | 3.39 |
| 19 | 0.00 |
| 20*** | 11.43 |
| 21 | 7.41 |
| 22 | 0.00 |
| 23 | 24.00 |

**Table S2.** *Measured at Night, during sleep

**Two nights no HR readings but sleep was measured effectively (HR was missing in 11.84%)

***Participant only choose to use ring during the night
